# Platelet-rich plasma (PRP) versus steroid as an intradiscal injection for lower back aches among degenerative disc disorder patient in India

**DOI:** 10.6026/973206300222630

**Published:** 2026-04-30

**Authors:** Jaideep Singh, Urmila Keshari, V Vignesh Rajan, Sivasamy Sivakumar, Varsha Manal

**Affiliations:** 1 Department of Anaesthesiology, Gandhi Medical College, Bhopal, Madhya Pradesh, India

**Keywords:** Platelet rich plasma (PRP), chronic radicular pain, degenerative disc disorder (DDD), visual analog scale (VAS), regenerative therapy

## Abstract

Degenerative disc disease (DDD) is a major cause of chronic low back pain and optimal minimally invasive treatment remains uncertain. Therefore, it is of interest to compare the efficacy of intradiscal platelet-rich plasma (PRP) versus steroid injections using Visual Analog Scale (VAS) scores. In this prospective study of 60 patients, participants were randomized to receive either triamcinolone or PRP injections weekly for three weeks, with follow-up at two and six months. Steroid injections provided greater short-term pain relief at two months, whereas PRP showed significantly superior pain reduction at six months with lower final VAS scores. Both modalities are effective in the short term, but PRP offers better sustained pain relief and may be preferred for long-term management of degenerative disc disease.

## Background:

Low back pain (LBP) is a widespread pain disorder affecting populations globally [1]. It has emerged as a significant public health concern and remains the leading cause of years lived with disability worldwide [22]. Its chronic nature and high prevalence pose substantial economic and healthcare burdens [3]. LBP resulting from intervertebral disc degeneration and lumbar disc herniation necessitates a multifaceted treatment approach, ranging from conservative management to surgical intervention [4, 5]. Degenerative disc disease (DDD) has been well established as a primary etiology of chronic LBP [6]. The intervertebral discs, characterized by limited vascularization, possess a suboptimal healing environment, predisposing them to progressive degeneration [7, 8-9]. While surgical intervention is often recommended for patients with symptomatic DDD unresponsive to conservative treatment and remains an option for refractory cases, it is associated with increased invasiveness and potential post-operative complications, including reduced lumbar range of motion and adjacent segment degeneration. There were also studies regarding mesenchymal stem cell therapy for DDD [10]. Furthermore, surgery is not without limitations. Consequently, there is a growing interest in regenerative medicine as a minimally invasive alternative for managing discogenic pain like lumbar radiculopathy with selective nerve root block by using sodium hyaluronate carboxymethyl cellulose solution [11, 12]. Epidural steroid injections (ESIs), with or without local anaesthetics, are a common non-surgical intervention employed to manage chronic radicular pain associated with DDD; however, their efficacy is often transient [13, 14]. These injections can be administered via several routes, including transforaminal, caudal, and interlaminar approaches. The transforaminal approach is often favoured due to its purported ability to deliver medication directly to the affected neural structures, potentially maximizing therapeutic effect while minimizing systemic exposure [15]. The choice of pharmacological agents for transforaminal injections has been a subject of ongoing debate. Corticosteroids, particularly triamcinolone, remain the gold standard [16], with other steroidal agents such as betamethasone, methylprednisolone, and etanercept also demonstrating efficacy [17]. Additionally, local anaesthetics such as lignocaine and bupivacaine, as well as adjuncts like sodium hyaluronate/carboxymethyl cellulose solution, have been explored for their therapeutic potential [18]. Among emerging regenerative therapeutic options, bone marrow aspirate and platelet-rich plasma (PRP) intradiscal injections have garnered attention due to their potential for disc repair and regeneration [19, 20]. PRP is an autologous concentrate of fibrin and growth factors derived from peripheral blood, prepared through centrifugation [21]. Its reparative properties have been documented in the treatment of collagen-based tissue injuries, including ligaments, cartilage, and tendons [19, 20]. Despite promising findings, comparative studies evaluating PRP and steroidal agents for the intradiscal route of injection remain limited. So far, only a limited number of studies have directly compared the outcomes of lumbar transforaminal injections using steroids and PRP [19, 20]. Therefore, it is of interest to determine their relative effectiveness in treating lower back pain related to degenerative disc disease.

## Materials and Methods:

This prospective comparative study was conducted at Gandhi Medical College, Bhopal. Sixty patients with lumbar degenerative disc disease were included. The commonly affected levels were L2-L3, L3-L4 and L4-L5. Patients were randomly divided into two groups. Baseline pain was assessed using the Visual Analog Scale (VAS). Group A received intradiscal triamcinolone injections. Injections were given once weekly for three weeks. Group B received intradiscal platelet-rich plasma (PRP). PRP was prepared from autologous blood. The injection schedule was identical to Group A. Pain relief was assessed at two and six months. VAS scores were recorded at each follow-up. Mean VAS scores were compared between both groups. Comparisons were made at baseline, two months and six months. Inclusion criteria included patients aged 35-55 years. All patients had MRI-confirmed lumbar disc disease. Chronic low back pain was present for more than six months. VAS score was greater than 4. Patients with prior lumbar surgery were excluded. Patients with infections, malignancy, or autoimmune disease were excluded. Patients with coagulopathy or pregnancy were excluded. Patients with steroid or PRP allergy were excluded. Patients with spinal deformity or prior fusion were excluded. Recent intradiscal injections within six months were excluded. Patients with severe neurological deficits were excluded. Data analysis was performed using JAMOVI version 2.6.13. Continuous variables were expressed as mean ± SD. Unpaired t-test was used for comparison. Normality was assessed using the Shapiro-Wilk test. Mann-Whitney U test was used for non-parametric data. Categorical variables were analyzed using Chi-square test. A p-value <0.05 was considered statistically significant

## Results:

A total of 60 patients were enrolled in the study and equally randomized into Group A (Steroid) and Group B (PRP), with 30 patients in each group. The age of the study population ranged from 35 to 55 years. The majority of patients in both groups were within the 46-50 years age category. The mean age in Group A was 46.07 ± 4.86 years, while in Group B it was 46.63 ± 4.50 years. There was no statistically significant difference in age distribution between the groups (p = 0.641) (Figure 1 - see PDF). In terms of gender distribution, females constituted a slightly higher proportion in both groups. Group A included 16 females (53.3%) and 14 males (46.7%), whereas Group B included 18 females (60.0%) and 12 males (40.0%). The difference in gender distribution between the groups was not statistically significant (p = 0.602) (Figure 2 - see PDF). At baseline, both groups had comparable VAS scores (Group A: 7.83 ± 1.12, Group B: 7.87 ± 0.9, p = 0.899). At 2 months, Group A showed a greater initial reduction (VAS: 6.03 ± 1.4) than Group B (VAS: 6.5 ± 1.01), through the difference was not statistically significant (p = 0.144). However, by 6 months, PRP demonstrated superior long-term efficacy, with a significant reduction in pain (VAS: 5.03 ± 1.27) compared to the steroid group (VAS: 6.47 ± 1.2, p < 0.001) (Table 1 - see PDF). Percentage improvement in VAS scores at 2 months was higher in the steroid group (22.99% ± 5%) than in the PRP group (17.41% ± 6%), but by 6 months, PRP showed significantly greater improvement (36.09% ± 7%) compared to steroids (17.37% ± 4%), with a p-value <0.001 (Table 2 - see PDF). This suggests that while steroids provide faster short-term relief, PRP offers superior long-term pain reduction, likely due to its regenerative properties, whereas steroid effects.

## Discussion:

In the present study, both platelet-rich plasma (PRP) and corticosteroid injections were effective in providing short-term pain relief in patients with degenerative disc disease. However, PRP demonstrated better long-term outcomes, with sustained reduction in pain scores at six months when compared to corticosteroids. The early improvement seen in both groups is likely related to their anti-inflammatory effects. Corticosteroids such as triamcinolone are known to suppress inflammatory mediators and provide rapid pain relief, but this effect is often temporary and tends to decline over time [16]. On the other hand, PRP contains multiple growth factors, including platelet-derived growth factor and transforming growth factor-β, which is involved in tissue healing and regeneration [21]. This may explain the more sustained effect seen with PRP. A similar trend was reported by Akeda *et al.* [2], who observed a significant reduction in VAS scores from 7.5±1.2 to 3.2±2.4 (p<0.01), with improvement maintained for up to six months. Our findings are in line with previous studies that have shown longer-lasting pain relief with PRP injections compared to corticosteroids [19, 20]. Several authors have reported meaningful reductions in VAS scores at mid- and long-term follow- up with PRP, supporting its use as a regenerative treatment option. In contrast, corticosteroid injections appear to be more useful for short-term symptom control, with limited long-term benefit [13, 14]. The difference in outcomes between the two treatments may be related to their mechanisms of action. PRP is thought to promote healing within the degenerated disc, whereas corticosteroids mainly reduce inflammation without addressing the underlying pathology. This could explain why the effects of PRP tend to persist longer. Recent study conducted by Mounisamy *et al.* [21] also showed better reduction in VAS scoring from 8 to 5 after using PRP. There are some limitations to this study. The sample size was relatively small, and the follow-up period was limited to six months. In addition, differences in PRP preparation techniques and the lack of standardization may influence the outcomes. Further studies with larger sample sizes, standardized protocols, and longer follow-up are needed to confirm these findings.

## Conclusion:

PRP and corticosteroid injections are effective for short-term pain relief in degenerative disc disease. PRP shows superior long-term efficacy with sustained pain reduction at six months, unlike corticosteroids whose effects decline over time. Larger studies with longer follow-up are required to standardize PRP protocols and confirm its role in long-term management.

## Advancement to knowledge:

This study provides comparative clinical evidence supporting the superior long-term efficacy of intradiscal platelet-rich plasma over corticosteroid injections in degenerative disc disease. The findings highlight a time-dependent therapeutic divergence, where corticosteroids offer transient anti-inflammatory effects, whereas PRP promotes sustained pain reduction, likely through growth factor- mediated regenerative pathways. These results contribute to the evolving understanding of biologic therapies in disc degeneration and support the integration of regenerative strategies into precision-based management of chronic low back pain.
